# Bioinformatic identification of connective tissue growth factor as an osteogenic protein within skeletal muscle

**DOI:** 10.14814/phy2.12255

**Published:** 2014-12-24

**Authors:** Steven J. Forrester, Keisuke Kawata, Hojun Lee, Ji‐Seok Kim, Kelly Sebzda, Tiffiny Butler, Vanessa R. Yingling, Joon‐Young Park

**Affiliations:** 1 Cardiovascular Genomics Laboratory, Department of Kinesiology, College of Public Health, Temple University, Philadelphia, Pennsylvania; 2 Department of Kinesiology, California State University, East BayHayward, California; 3 Cardiovascular Research Center, School of Medicine, Temple UniversityPhiladelphia, Pennsylvania

**Keywords:** Calorie Restriction, CTGF, exercise, myokine

## Abstract

Aging is associated with increasing incidence of osteoporosis; a skeletal disorder characterized by compromised bone strength that may predispose patients to an increased risk of fracture. It is imperative to identify novel ways in which to attenuate such declines in the functional properties of bone. The purpose of this study was to identify, through in silico, in vitro, and in vivo approaches, a protein secreted from skeletal muscle that is putatively involved in bone formation. We performed a functional annotation bioinformatic analysis of human skeletal muscle‐derived secretomes (*n* = 319) using DAVID software. Cross‐referencing was conducted using OMIM, Unigene, UniProt, GEO, and CGAP databases. Signal peptides and transmembrane residues were analyzed using SignalP and TMHMM software. To further investigate functionality of the identified protein, L6 and C2C12 myotubes were grown for in vitro analysis. C2C12 myotubes were subjected to 16 h of glucose deprivation (GD) prior to analysis. In vivo experiments included analysis of 6‐week calorie restricted (CR) rat muscle samples. Bioinformatic analysis yielded 15 genes of interest. GEO dataset analysis identified BMP5, COL1A2, CTGF, MGP, MMP2, and SPARC as potential targets for further processing. Following TMHMM and SignalP processing, CTGF was chosen as a candidate gene. CTGF expression level was increased during L6 myoblast differentiation (*P *<**0.01). C2C12 myotubes showed no change in response to GD. Rat soleus muscle samples exhibited an increase in CTGF expression (*n* = 16) in response to CR (35%) (*P *<**0.05). CTGF was identified as a skeletal muscle expressed protein through bioinformatic analysis of skeletal muscle‐derived secretomes and in vitro/in vivo analysis. Future study is needed to determine the role of muscle‐derived CTGF in bone formation and remodeling processes.

## Abstract (precis)

In this study, we explore the method of bioinformatic analysis, coupled with in vitro and in vivo investigation, to identify a new skeletal muscle‐derived protein with osteogenic properties. CTGF is expressed in young, healthy skeletal muscle, and this expression is increased with calorie restriction. Muscular secretion of CTGF might play an osteogenic role in maintaining bone health.

## Introduction

Bone research is a widely expanding area of focus in the world of experimental physiology, and within this subject the utmost importance is placed on age and disease‐related declines associated with bone structure and function (Demontiero et al. 2012). Maintaining bone health is vital to quality of life as it aids in functions such as locomotion, protection of internal organs, maintaining posture, and serving as a reservoir for calcium (DiGirolamo et al. 2013). Throughout life, bone is continually remodeling itself, adapting to outside environments by either building itself up with the help from osteoblasts or breaking itself down through the actions of osteoclasts (Chan and Duque 2002; Demontiero et al. 2012). Inevitably due to old age and disease, bones suffer from decreases in bone mineral density, strength, and increased fragility. Ultimately, these complications result in osteoporosis within the elderly population. As well, decreased bone health is associated with a decrease in the quality of life of the affected individual (Cooper 1997). Current topics under investigation in relation to bone include nutritional status, hormones, genetic background, and physical activity (Chan and Duque 2002; Demontiero et al. 2012; DiGirolamo et al. 2013).

Research has suggested that skeletal muscle, while being a distinct separate tissue, shares a close relationship with bone. The development of skeletal muscle along with bone is vital for an organism to survive. Pertinent to our study is the importance of skeletal muscle in physical activity and nutrition. Physical activity has been observed to attenuate age‐related declines in muscle function and is associated with increased quality of life in the elderly. As well, skeletal muscle plays a large role in mediating the adaptations experienced with exercise training such as increased hypertrophy and increased strength (Hunter et al. 2004). Increased strength allows for more mechanical loading leading to increased mechanical stress on bone, which facilitates bone adaptations such as increased density and mass (Layne and Nelson 1999). Along with the classical adaptations, exercise has allowed the study of skeletal muscle as a secretory organ in recent years (Pedersen 2011; Pedersen and Febbraio 2012). Investigation has led to the observation that skeletal muscle, in response to physical activity or other stimuli, is capable of releasing a secretome containing myokines which can exert their effects in an autocrine, paracrine, or endocrine manner. Since little is known of the clinical significance derived from myokines, further research is warranted in this field in order to elucidate novel myokines, which may be useful in therapeutics.

Recently, calorie restriction (CR) has been a focal point of aging research due to its beneficial effects on cell health (Lin et al. 2002; Cohen et al. 2004). From a skeletal muscle perspective, CR within mice has yielded such findings as decreased DNA damage, increased mitochondrial gene expression, and increased NAD^+^‐dependent deactylases, and it is hypothesized that these adaptations may contribute to the beneficial effects of CR in prolonging life (Civitarese et al. 2007). In relation to bone, however, CR studies have generated varying results in modulating bone properties (Villareal et al. 2006; Redman et al. 2008; Tatsumi et al. 2008). Specifically, Tatsumi et al. (2008) analyzed life‐long CR versus Ad libitum feeding in mice and rats, and discovered that while CR might be detrimental to bone formation in the maturational stages of life, CR appeared to decrease age‐associated loss of bone mass in the postmaturational stage in both species. Due to the relationship between skeletal muscle and bone it would be intriguing to observe the multifaceted connections and possible beneficial outcomes between these two tissues when subjected to CR.

Playing a role in bone morphogenic properties is the cytokine connective tissue growth factor (CTGF). CTGF is a 38 kDa extracellular matrix protein involved in numerous physiological processes such as chondrogenesis, fibrosis, osteogenesis, and angiogenesis (Safadi et al. 2003; Kubota and Takigawa 2007, 2011; Wang et al. 2009; Arnott et al. 2011). Of central importance to this study, CTGF has been observed to be involved in numerous bone formation processes such as endochondral and intramembranous ossification (Arnott et al. 2011), osteoblast differentiation and proliferation (Safadi et al. 2003), and osteogenic differentiation of mesenchymal stem cells (Wang et al. 2009). While information on skeletal tissue and CTGF is numerous, information relating to CTGF's expression within skeletal muscle is inconclusive and is of value to the medical field.

The purpose of the current study was to identify, through bioinformatic analyses coupled with in vitro and in vivo approaches, a skeletal muscle‐derived myokine involved in osteogenic processes. Through identification of an osteogenic myokine, the relationship between muscle and bone becomes unequivocal and is a vital step in developing methods to treat age and disease‐related declines in bone health.

## Methods

### Animals

Use of animals was approved by Institutional Animal Care and Use Committee at Temple University. Full animal procedure is mentioned as discussed in Butler et al. (2013), but briefly, sixteen female Sprague‐Dawley rats, 9 weeks of age, were assigned to either a control group (Con) (*n* = 8), or a calorie restriction group (CR) (*n* = 8). Control rats were allowed to eat ad libitum. CR rats were fed 70% of control ad libitum food amount for 6 weeks with a micronutrient replete food preparation (D10012G, Research Diets, Inc., New Brunswick, NJ) Food amount was adjusted daily based on the average food intake of control animals. After 6 weeks of CR, all animals were sacrificed using CO_2_ inhalation. Muscle samples were isolated, weighed, and immediately frozen at −80°C until further use. Animals were housed using a 12 h light/dark cycle with consistent temperature (21–31°C) and humidity (58–60%).

### Secretome acquisition and analysis

To identify a potential myokine, an initial functional annotation bioinformatic analysis was performed using human skeletal muscle‐derived secretomes (*n* = 319) previously identified through a genome‐wide prediction study (Bortoluzzi et al. 2006). Bioinformatic analysis was conducted on the basis of identifying proteins involved in multiple processes of bone development. Each gene was categorized into a total of seventeen distinct bone processes. These seventeen processes are listed in Table 1. Analysis was conducted through the use of the Database for Annotation, Visualization and Integrated Discovery (DAVID) software v 6.7 (NCBI, NIH) (Huang et al. 2008, 2009). DAVID is a widely recognized bioinformatic tool developed to provide insight into the functional complexity of large gene lists. Functions of DAVID include enriched biological themes, functionally related gene groups, interacting proteins, gene‐disease associations, protein functional domains and motifs, molecular pathway information regarding single genes and gene clusters (Huang et al. 2008, 2009). Subsequent examination was conducted using OMIM, Unigene, UniProt, Cancer Genome Anatomy Project (CGAP), and the GEO database (Boguski and Schuler 1995; Schuler et al. 1996; Schuler 1997; Edgar et al. 2002; Pontius et al. 2003; Wheeler et al. 2003; The UniProt Consortium 2013). After collection of bone characteristic data for each gene, genes with the greatest number of bone‐associated functions were analyzed using GeoData sets to further delineate specific candidate genes. GEO datasets (http://www.ncbi.nlm.nih.gov/gds) were analyzed for muscle gene expression profiles relating to exercise, or muscle‐stimulated interventions. Gene expression results were quantitated and filtered for selection before narrowing choices of candidate genes.

**Table 1.** The osteogenic properties that were analyzed during DAVID functional annotation and cross‐referencing

Osteogenic properties	
Bone density	CGAP: normal bone
Bone density osteoporosis	CGAP: bone marrow
Bone development	Intramembrane ossification
Bone marrow	Ossification
Bone neoplasia	Osteoarthritis
Bone trabeculae formation	Regulation of ossification
Cartiage development (Endochondral bone morphogenesis)	Skeletal system development Unigene: bone Unigene: bone marrow

### Algorithmic prediction of transmembrane helices and signal peptides

Selected genes were analyzed through the use of TMHMM and SignalP 4.1 software (Sonnhammer et al. 1998; Krogh et al. 2001; Petersen et al. 2011). An extensive understanding of the TMHMM and SignalP 4.1 can be found through the work of Krogh et al. (2001) and Petersen et al. (2011). Briefly, TMHMM is the software used to predict transmembrane helices (TMHs) within an amino acid (AA) sequence, and SignalP is used to identify signal peptides within an AA sequence. After analysis of an AA sequence, TMHMM yields statistics and indices relevant to the probability of a TMH. Based on the AA sequence, TMHMM predicts a certain number of TMHs for a given AA sequence, along with the expected number of AAs within a TMH. If the number of AA within the predicted TMH is larger than 18 AAs, the likelihood of a transmembrane protein, or a signal peptide, is high. To distinguish the possibility of a signal peptide and not a TMH, the expected number of AAs in the TMH within the first 60 AAs of a given gene is used to measure the presence of a possible signal peptide; signal peptides are generally located on the N‐terminus side of an AA sequence. A moderately low to high value indicates a TMH in the N‐terminus is likely to be a signal peptide and further prediction software should be run. Lastly, total probability of N‐in (AAs that sit inside the membrane) is a measure of the probability that the N‐terminus is on the cytoplasmic side of the cell membrane. Results are plotted as the probability of a residue sitting in helix, inside, or outside summed over all possible model paths. Graphical output illustrates probability (*y*‐axis) for each individual AA (*x*‐axis) within a protein. Between values 1 and 1.2 on the *y*‐axis, N‐best prediction is illustrated. N‐best prediction is an algorithm used to find the most probable topology of a membrane protein (Sonnhammer et al. 1998; Krogh et al. 2001). Ultimately, results of TMHMM analysis will indicate the presence of a transmembrane helix within a given protein.

SignalP is an algorithmic tool used to predict if an AA sequence of a given protein contains a signal peptide as an indication of secretion. Through analysis, three separate scores are generated for each specific AA sequence. The first of the three scores is the C‐score, which is a raw cleavage site score used to distinguish the presence of a signal peptide cleavage within the AA sequence. The score is the greatest at the position immediately following the cleavage site. The S‐score is a means to recognize positions within signal peptides from positions in the remaining AA sequence of a given protein. Additionally, the S‐score differentiates between proteins with signal peptides, and those without. Finally, the Y‐score is a combination of the C‐score and slope of the steepest S‐score, providing a more thorough prediction of cleavage site as opposed to C‐score alone which can have multiple peaks. Graphical output is created by illustrating all three scores for each AA within a protein. Additionally, because signal peptides are generally found on the N‐terminus of an amino acid sequence, SignalP graphical output illustrates the first 70 AAs of a given sequence. Numerical data is often given with graphical output, but intersection between scores above threshold (D‐cutoff) is a characteristic of secreted proteins. D‐cutoff is optimized based upon Matthews Correlation Coefficient. This results in low sensitivity to signal peptides, thus deterring false‐positive identification (Petersen et al. 2011). Overall, combining TMHMM and SignalP results, if a given gene is predicted to have a TMH, or a TMH and a signal peptide, it is considered to be a membrane anchored protein. If the gene contains no TMH, but does contain a signal peptide, then it is considered a secreted protein.

After evaluation, all results were collected and analyzed in order to elucidate one potential candidate gene for downstream processing for in vitro and in vivo applications.

### Cell culture and protein extraction

L6 and C2C12 myoblast cells were obtained from American Type Culture Condition and grown in Dulbecco's modified Eagle Medium (DMEM) supplemented with 10% Fetal Bovine Serum (GIBCO) and 1% penicillin/streptomycin in a 5% CO_2_ incubator at 37°C. Upon confluency (90–95%), myoblasts were subjected to starvation‐mediated differentiation using differentiation media (DM) consisting of DMEM supplemented with 2% horse serum (Invitrogen) and 1% penicillin/streptomycin. L6 myoblasts were harvested on days 0, 1, 2, and 3 of differentiation for Western blot analysis. C2C12 myoblasts were differentiated for 5 days whereupon they were subjected to glucose deprivation by replacing differentiation media with glucose‐free DMEM supplemented with 2% Horse Serum for 16 h which were then harvested. Cell lysates from both L6 and C2C12 myoblasts were prepared as follows. Cells were washed three times with chilled DPBS and lysed using cold RIPA buffer (10 mmol/L Tris·HCl, 5 mmol/L EDTA, 150 mmol/L NaCl, 1% Triton X‐100, 0.1% SDS, 1% deoxycholate, pH 7.5) including protease and phosphatase inhibitors. Samples were then centrifuged (1600 g for 15 min at 4°C). The supernatant was collected and protein concentration was determined using the Bradford assay.

To prepare protein sample from in vivo tissue, rat skeletal muscle was weighed and submerged in RIPA buffer (1:10 w/v) containing protease and phosphatase inhibitors. Samples were homogenized (POLLY TRON, PT 2100) and subsequently subjected to sonification. Samples were then centrifuged (1600 g for 15 min at 4°C). The supernatant was collected and the Bradford assay was performed to determine protein concentration.

### Immunoblotting

After Bradford analysis, collected samples were subjected to SDS‐PAGE using 10% polyacrylamide gels. Electrophoresis was conducted at 120V, and proteins were transferred to Immobilon‐P membranes (Millipore, Billerica, MA) (100 mA, 25V, 120 min). After transfer, membranes were stained in Ponceau S (Sigma‐Aldrich, St. Louis, MO) to determine equal loading throughout. As well, for L6 myoblasts *α*‐tubulin was used to equalize protein loading. Membranes were then washed and blocked in Tris‐buffered saline containing 0.05% Tween20 (TBST) and 5% nonfat dry milk at room temperature for 15 min. After blocking, membranes were incubated overnight with a primary antibody at 4°C. Primary antibodies were as follows: goat polyclonal CTGF (Santa Cruz Biotechnology, Santa Cruz, CA), and mouse monoclonal *α*‐tubulin (Sigma‐Aldrich). Membranes were then washed three times in TBST and incubated with appropriate secondary antibody (peroxidase‐conjugated bovine anti‐goat IgG, or anti‐mouse IgG) (Jackson ImmunoResearch Labs, West Grove, PA) for 1 h at room temperature. Membranes were washed three times in TBST and protein expression was observed using chemiluminescence visualization (Thermo Fisher Scientific Inc., Waltham, MA).

### Statistics

Results are presented as mean ± SEM, unless indicated otherwise, for a minimum of three independent experiments in triplicate. All comparisons were made against a control condition using either one‐way ANOVA with a Turkey's post hoc test or Student's t‐test depending upon the number of independent variables tested. The level of significance was set at *P *<**0.05.

## Results

### In silico analysis of human‐derived myoblast secretome

**Figure 1.**
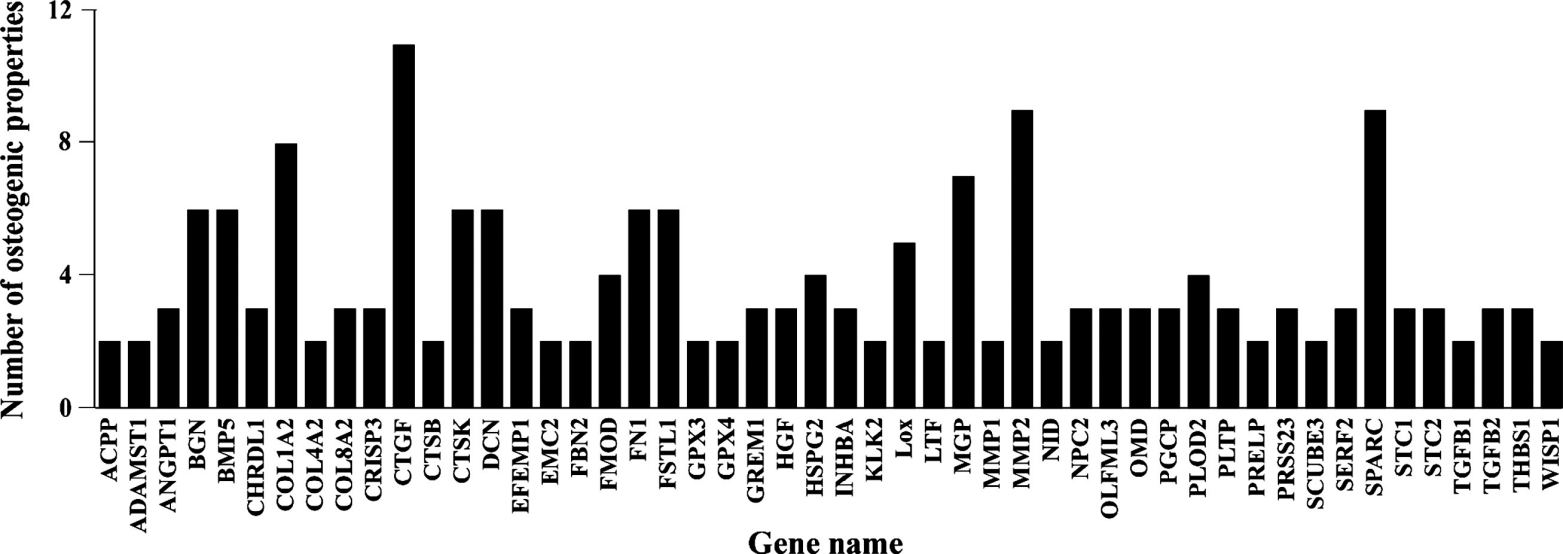
Graphical output illustrating top 50 genes identified by in silico analysis.

To identify a potential myokine involved in bone functionality, DAVID, along with UniProt, Unigene, CGAP, and OMIM, was used to analyze human myoblast secretomes. Using these tools, we analyzed 319 genes for 17 distinct osteogenic functions (Table 1). Figure 1 displays the top 50 results from analysis. A threshold of 4 osteogenic functions was instated to substantiate further analysis of candidate genes. We rationalized using 4 as a threshold that allowed us to discern between genes that could be potential false positives, and genes illustrating a trend in osteogenic function.

### GEO data set analysis of selected genes

Fifteen genes were identified for further analysis and were subsequently analyzed for GEO data sets involving exercise and skeletal muscle specific intervention. (Table 2). Through data set analysis, three genes were identified as having significant increases in gene expression in response to exercise or intervention (Table 2). These three genes were BMP5 (bone morphogenic protein 5), CTGF (connective tissue growth factor), and SPARC (osteonectin, secreted protein, acidic, cysteine‐rich).

**Table 2.** GEO dataset analysis of potential skeletal muscle genes

Gene	Hits	GEO data	Subject	Method	Pre (A.U.)	Post (A.U.)
BGN	6	GDS894	Human	Resistance exercise	1100.0 ± 60.0	1618.0 ± 605.0
BMP5	6	GDS4035	Rat	Aerobic exercise in low A.C. rat	4.6 ± 0.1	4.9 ± 0.1*
COL1A2	8	GDS2740	Human	24 h lengthening contraction	46.7 ± 14.2	86.6 ± 19.5
CTGF	11	GDS894	Human	6 h after resistance exercise	90.3 ± 2.1	133.8 ± 17.7*
CTSK	6	GDS894	Human	6 h after resistance exercise	116.8 ± 5.3	191.8 ± 77.4
DCN	6	GDS2740	Human	3 h after lengthening contraction	38.4 ± 8.9	37.7 ± 10.3
FMOD	4	GDS2730	Human	6 h after lengthening contraction	46.0 ± 3.4	33.5 ± 6.4
FN1	6	GDS894	Human	Resistance exercise	424.4 ± 32.1	711.7 ± 324.9
FSTL1	6	GDS4035	Rat	Aerobic exercise in low A.C rat	7.8 ± 0.3	8.6 ± 0.2
HSPG2	4	GDS925	Mouse	Myotube starvation	1554.7 ± 135.1	1019.2 ± 88.4
LOX	5	GDS2740	Human	3 h after lengthening contraction	40.4 ± 13.6	77.6 ± 4.2
MGP	7	GDS2740	Human	6 h after lengthening contraction	343.3 ± 27.2	285.6 ± 50.6
MMP2	9	GDS2740	Human	24 h after lengthening contraction	131.2 ± 24.1	38.3 ± 3.4*
PLOD2	4	GDS925	Mouse	Myotube starvation	787.4 ± 49.8	585.6 ± 59.5*
SPARC	9	GDS915	Human	Aerobic exercise	1007.1 ± 46.1	3376.6 ± 500.6*

Results are presented as mean ± SEM.

A.C., Aerobic Capacity; A.U., Arbitrary Units.

* *P* ≤ 0.05.

GEO data sets were chosen by searching for key factors related to exercise training such as “exercise”, “skeletal muscle”, “aerobic or endurance training”, and “anaerobic or resistance training”, along with the gene name. GDS4035 from Bye et al. (2008) analyzed the exercise effect on rat soleus muscle samples from high and low capacity runner populations. In the low aerobic capacity runner muscle samples BMP5 increased by 6.5% (4.6 ± 0.1 vs. 4.9 ± 0.1) (*P *<**0.05), in response to aerobic exercise (Bye et al. 2008).

GDS894 analyzed skeletal muscle response to exercise and circadian rhythms (Zambon et al. 2003). mRNA was extracted from human vastus lateralis muscle biopsy samples 6 h after an acute bout of isotonic resistance exercise. Resistance exercise resulted in an increase in CTGF mRNA expression (90.3 ± 2.1 vs. 133.8 ± 17.7) (*P *< 0.05) compared to nonxercise group. Lastly, GDS915, from the group of Hittel et al. (2005), examined the effects of aerobic training in skeletal muscle gene expression from overweight males with metabolic syndrome. Muscle biopsies were excised from the vastus lateralis. SPARC gene expression increased compared to control as a result of aerobic exercise training (2,352%) (1007.1 ± 46.1 vs. 3376.6 ± 500.6) (*P *<**0.05) (Hittel et al. 2005).

**Figure 2.**
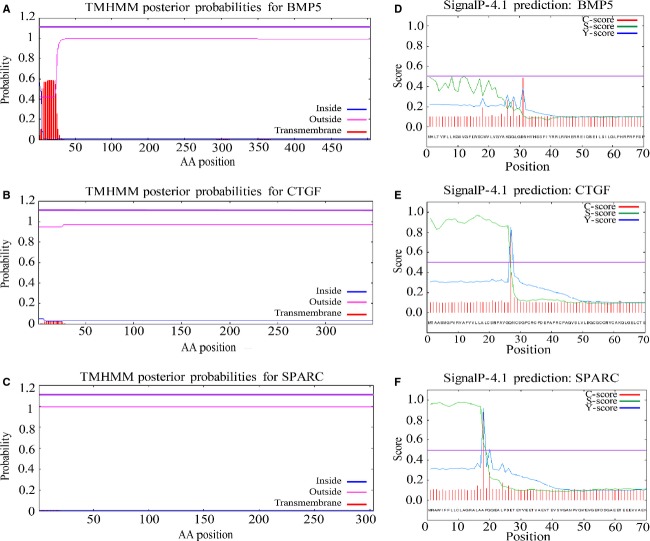
THMHH (A–C) and SignalP 4.1 (D–F) results of 3 individual genes. Analysis was conducted using entire amino acid sequence for each gene. (A and D) BMP5, (B and E) CTGF, and (C and F) SPARC. Plots for TMHMM are presented as probability (*y*‐axis) of an amino acid (*x*‐axis) residue sitting in helix, inside, or outside summed over all possible model paths. N‐best prediction threshold is scored between 1 and 1.2 (purple line). Transmembrane represents probability on an amino acid sitting within a transmembrane helix. Inside represents probability of an AA residue sitting within the cytoplasmic side of a membrane. Outside depicts probability of an AA residue resting within extracellular space. SignalP results graphed using C‐score, S‐score, and Y‐score. Default‐cutoff (D‐cutoff) was set at 0.5 (purple line). Genes with graphical intersections above D‐cutoff are considered to contain signal peptides. CTGF and SPARC were predicted to contain signal peptides without the presence of a TMH, whereas BMP5 was predicted not to contain either a TMH or a signal peptide.

For further details with regard to type of intervention, significance, and subject, Table 2 lists all relevant information including GEO data set codes for each individual gene. After GEO data set analysis, candidate genes were narrowed based on results from both DAVID and GEO data set results. CTGF, BMP5, and SPARC were chosen to process with SignalP 4.1 and TMHMM software (Center for Biological Sequence Analysis, Kongens Lyngby, Denmark) (Fig. 2).

### TMHMM and SignalP 4.1 analysis

Of the three genes analyzed, none showed the presence of a transmembrane helix. BMP5 was the closest to containing a possible transmembrane helici by scoring a 12.09 and 12.08 in the expected number of AA's in TMHs and the expected number within the first 60 AAs, respectively. Total probability of N‐in was 0.57. CTGF scored 0.47 and 0.46 in the expected number of AAs in TMHs and the expected number within first 60 AAs, respectively, as well as having a total probability of N‐in of 0.05. Lastly, SPARC scored 0.007 and 0.007 in the expected number of AAs in TMHs and the expected number within first 60 AAs, respectively. Additionally, the total probability of N‐in for SPARC was 0.006. Figure 2(A–C) represents the results of TMHMM software analysis and shows plots of all proteins. SignalP 4.1 provided intriguing results as BMP5 was deemed to be without a signal peptide (Fig. 2D). Thus, eliminating BMP5 as a candidate gene. From our bioinformatic analysis, we narrowed our scope from 319 potential genes down to 2. CTGF was predicted to contain a signal peptide between glycine 26 and glutamine 27, and SPARC between residues Alanine 16 and Alanine 17 (Fig. 2D–F). Based on in silico results from each step of the analysis, we chose CTGF as our candidate gene for in vitro and in vivo testing. CTGF scored the highest value from DAVID and database cross‐referencing, showed significant gene expression changes (*P *<**0.05) within skeletal muscle in response to resistance exercise (Table 2), and was determined to contain a signal peptide along with no TMHs using TMHMM and SignalP 4.1 (Fig. 2B and E), respectively.

### CTGF increases during myoblast differentiation and is unaffected by glucose deprivation

**Figure 3.**
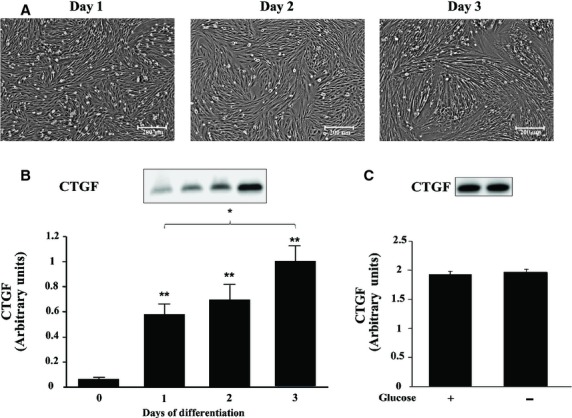
L6 and C2C12myoblast cell culture analysis. (A) Pictures L6 myoblasts during days 1, 2, and 3 of differentiation. (B) CTGF expression of CTGF during 3 days of differentiation. Equal loading was verified using Ponceau S staining and *α*‐tubulin. (C) C2C12 myoblasts were differentiated for 5 days and were subjected to glucose deprivation for 16 h. *P <**0.05, **P <**0.01 versus Day 0.

To determine if CTGF was a skeletal muscle expressed protein in vitro, we performed complementary in vitro experiments using L6 and C2C12 myoblast cell lines. Each day of myoblast differentiation resulted in significant increases in CTGF expression compared to day 0 (*P *<**0.01) with the greatest increase occurring on day 3 (~1500%) (Fig. 3B).

We further investigated how CTGF expression might be affected depending upon different stimuli. We subjected C2C12 myotubes, which were differentiated for 5 days, to glucose deprivation for 16 h in order to mimic starvation conditions. To our surprise, CTGF expression was not changed between control and glucose deprivation groups (*P > *0.05) (Fig. 3C).

### CTGF is expressed in vivo and is increased in response to calorie restriction

**Figure 4.**
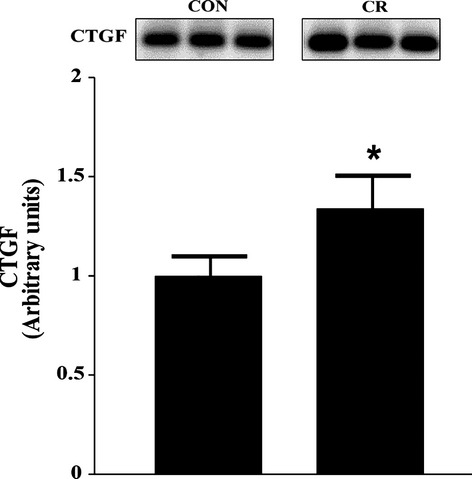
CTGF protein expression within rat soleus muscle after 6 weeks of caloric restriction (*n* = 16). A. CTGF expression between control and caloric restriction groups. Equal loading was verified using Ponceau S staining. *P <**0.05

Based on our findings of CTGF protein expression in vitro, we next examined CTGF expression at the tissue level. Sprague‐Dawley rats were subjected to a 30% CR for 6 weeks. Afterwards, we measured CTGF expression within the soleus muscle from both CON and CR groups. CTGF protein expression increased by 35% in the CR group compared to CON groups (0.75 ± 0.08 vs. 1.01 ± 0.13) (*P < *0.05*)* (Fig. 4).

## Discussion

Starting from a predicted myoblast secretome, we have shown through in silico, in vitro, and in vivo approaches that CTGF is expressed in young, healthy skeletal muscle. These results give validation for using a simple bioinformatic method to identify new myokines within the skeletal muscle.

Kivela et al. (2007) originally identified CTGF as a human skeletal muscle protein using a single bout of high mechanical loading imposed on the quadriceps. However, CTGF expression was neither clear in this study nor significant; indicating need for further investigation (Kivela et al. 2007). As well, CTGF has recently been observed to increase in skeletal muscle from dystrophic mice (Morales et al. 2013). Again however, CTGF expression in this study stemmed from a diseased mouse model, and furthermore, wild‐type levels of CTGF were scarcely expressed. CTGF is thought to be a positive modulator of fibrosis, and thus is constantly under scrutiny as a detrimental protein. Due to preconceived notions, investigations have ignored the potential view of CTGF as a positive regulator of physiological function. To the best of our knowledge, we are the first to show clear CTGF expression within healthy skeletal muscle, as well as an increase in protein expression during CR.

Along with exciting findings from our CR model, we observed increased CTGF expression throughout myotube differentiation in L6 skeletal muscle cells. The signal peptide contained within CTGF might play a vital role in skeletal muscle‐derived CTGF. Increased CTGF expression in young myoblasts could facilitate increased secretion of this protein to the extracellular matrix where it may communicate with bone. As well, due to the profound levels of CTGF observed during myotube formation, especially on day 3, we believe CTGF to be vital to skeletal muscle development and function. Supporting this notion, and during the time of manuscript submission, Nishida and colleagues published a magnificent finding showing CTGF is vital for myotube differentiation in C2C12 cells, thus providing initial physiological evidence of a function for CTGF within skeletal muscle (Nishida et al. 2014). Coupling our findings and the findings of Nishida, there is increased interest into the physiological aspect of CTGF in skeletal muscle and further research is needed to elucidate the exact role CTGF plays in skeletal muscle development and maintenance. Recently, Capparelli et al. (2012) demonstrated a unique role of CTGF in regulating cellular function within fibroblasts. Fibroblasts overexpressing CTGF showed increased levels of both autophagy and mitophagy markers microtubule‐associated protein 1A/1B‐light chain 3 (LC3), and BCL2/Adenovirus E1B 19 kDa Interacting Protein 3(BNIP3), respectively (Capparelli et al. 2012). Mitochondrial activity has been observed to be vital for differentiating myoblasts, and increased rates of cell proliferation and cell differentiation among myoblasts are characterized by increased rates of cell turnover and morphology (Rochard et al. 2000; Kim et al. 2013). While optimistic and not of the scope of this paper, functions of CTGF in skeletal muscle are scarce, and future studies might provide a link between CTGF and mitophagy within the skeletal muscle, especially in the wake of the newly defined myogenic role of CTGF (Nishida et al. 2014).

### Physiological implications of CTGF and clinical relevance

While it is true that increased CTGF expression is correlated with fibrosis, CTGF is a positive regulator of bone function; acting as the key factor in bone formation, specifically endochondral bone formation (Arnott et al. 2011). CTGF has been shown to be vital, having influences in major bone morphogenesis properties, including osteogenic differentiation of mesenchymal stem cells, osteoblast proliferation and differentiation, and overall bone formation in vivo (Safadi et al. 2003; Kubota and Takigawa 2007; Arnott et al. 2011). The wide range in functions and capabilities of CTGF spurs a unique idea that location of CTGF expression might play a secondary role to the time in development at which CTGF is expressed. Relative expression of CTGF from young skeletal muscle might be indicative of a positive physiological response to skeletal muscle development, whereas CTGF expression in aged skeletal muscle might be indicative of a fibrotic pathophysiological response, as increased skeletal muscle fibrosis is associated with aging (Brack et al. 2007). Additionally, increased CTGF expression with age contributes to skeletal muscle senescence (Du et al. 2014). Due to the secretory nature of CTGF, increased CTGF expression from developing skeletal muscle could play a role in overall bone development and health, especially in response to an adaptive stimulus such as CR or exercise. These are warranted topics in the pursuit of distinguishing the specific role of CTGF within skeletal muscle.

Another point in trying to distinguish the role of secreted skeletal muscle CTGF is the mechanism by which it is distributed throughout the body; whether it be through endocrine or paracrine mechanisms. Due to the close proximity of skeletal muscle and bone, we believe skeletal muscle‐derived CTGF to act through a paracrine mechanism in inducing physiological effects on neighboring bone. This can be illustrated by the fact that C57BL/6J mice undergoing jump training have significant increases in periosteal bone formation within the tibia, thus showing an area specific increase in response to stress (Kodama et al. 2000). In this case, if we were to believe skeletal muscle‐derived CTGF played a role in bone formation, we would hypothesize this to be more of a site‐specific response of skeletal muscle on bone. Even in the setting of CR, we believe systemic effects of CR on skeletal muscle result in local and site‐specific communication between bone and skeletal muscle. However, we do not rule out the possibility of an endocrine effect, as CR does result in systemic changes. Skeletal muscle‐derived CTGF could undergo secretion into the bloodstream, whereupon it acts on other tissues and organs.

It is well‐known that bone health is at an optimum in the early stages of life compared to later. Thus, if CTGF acts as a myokine which is capable of influencing bone homeostasis, then investigation should be conducted on a younger population. As well, moderate (30–40%) CR has been observed recently to be a positive modulator, increasing bone density and mechanical strength in postpubescent rodents (Butler 2013). From our experiment, we show increased CTGF expression in response to a 6‐week 30% CR diet. Recently, CR was observed to increase serum osteocalcin in rats, correlating to increased trabecular bone volume in the lumbar spine compared to control (Joshi et al. 2011). Additionally, CR promoted increased bone functional properties, including lager bone area, cortical area, and moment of inertia (Butler et al. 2013). In relation to skeletal muscle, and in support of a beneficial effect for CR, short term CR in mice promotes enhanced function of skeletal muscle stem cells in both young and old mice (Cerletti et al. 2012). Enhanced stem cell activity in mice undergoing CR promoted accelerated muscle regeneration to injury. The findings illustrate the beneficial physiological consequences of CR in skeletal muscle and provide further evidence and support to an enhanced relationship between skeletal muscle and bone in the setting of CR. Thus, it is plausible that CTGF from skeletal muscle might play a regulatory role in the adaptive response of bone to caloric restriction. Future studies are warranted on this topic.

## Limitations

The present study has several limitations. The aim of the current study was to identify a new myokine through a bioinformatic approach using a predicted myoblast secretome. Due to already discovered myokines involved in osteogenesis, we had to make some exclusions. Within our data, IGF‐1 and IL‐8 scored above threshold for osteogenic function (6 and 4, respectively). However, because we were looking for a new myokine, we excluded these two genes, as they have been previously investigated (Akerstrom et al. 2005; Hamrick 2011). Additionally, the predicted myoblast secretome did not include IL‐6 as a secreted protein. This could be due to multiple reasons, but as stated, this was an algorithm‐based human myoblast secretome. Therefore, it could be possible that IL‐6 did not meet certain criteria to be incorporated into the predicted secretome. Even if IL‐6 was included into the predicted secretome, we would have excluded it because it is already known as a myokine (Pedersen and Febbraio 2012). Another limitation could have been the use of CR as an experimental variable. Even though CR provided a positive outcome in regulating CTGF expression, a more preferential modality could be the use of resistance exercise. Currently, there is extensive evidence of increased maintenance of bone health and function in response to resistance exercise (Layne and Nelson 1999; Kodama et al. 2000). Thus, it would appear to be a useful stimulus to measure a skeletal muscle‐secreted protein involved in bone homeostasis. Another limitation we noticed comes from within bioinformatic data, and specifically the GEO data sets mentioned. The study design of these experiments called for isolation of muscle mRNA either hours after acute training bouts, or after weeks of exercise training. We speculate that CTGF to be a short‐term response gene as opposed to an adaptive one. It could be most beneficial to measure gene expression right after the cessation of training where gene expression levels are predicted to significantly increase as opposed to hours after. Future studies are aimed at using exercise as a stimulus that may provide further insights into skeletal muscle regulation of CTGF expression.

Overall, through in silico, in vitro, and in vivo approaches, we provide compelling evidence of a novel myokine. CTGF, while having extensive functions within the bone, is a skeletal muscle expressed protein that is associated with myoblast differentiation and CR. Future experimentation is warranted in the hope of supplying insight to CTGF as a potential therapeutic for populations suffering from poor bone health. Moreover, we wish to demonstrate the feasibility of using bioinformatic data analysis in studying muscle secretomes. We believe that it is an indispensable tool for scientists, allowing for exponential growth in knowledge. With rapid acquisition of knowledge, we may be better equipped to help improve the quality of life for all individuals.

## Conflict of Interest

None declared.

## References

[b1] Akerstrom T. Steensberg A. Keller P. Keller C. Penkowa M. Pedersen B. K. 2005 Exercise induces interleukin‐8 expression in human skeletal muscle. J. Physiol.; 563:507-516. 15618276 10.1113/jphysiol.2004.077610 PMC1665593

[b2] Arnott J. A. Lambi A. G. Mundy C. Hendesi H. Pixley R. A. Owen T. A. 2011 The role of connective tissue growth factor (CTGF/CCN2) in skeletogenesis. Crit. Rev. Eukaryot. Gene Expr.; 21:43-69. 21967332 10.1615/critreveukargeneexpr.v21.i1.40 PMC3357314

[b3] Boguski M. S. Schuler G. D. 1995 ESTablishing a human transcript map. Nat. Genet.; 10:369-371. 7670480 10.1038/ng0895-369

[b4] Bortoluzzi S. Scannapieco P. Cestaro A. Danieli G. A. Schiaffino S. 2006 Computational reconstruction of the human skeletal muscle secretome. Proteins; 62:776-792. 16342272 10.1002/prot.20803

[b5] Brack A. S. Conboy M. J. Roy S. Lee M. Kuo C. J. Keller C. 2007 Increased Wnt signaling during aging alters muscle stem cell fate and increases fibrosis. Science; 317:807-810. 17690295 10.1126/science.1144090

[b6] Butler T. 2013 The Effects of Post Pubertal Food Restriction on Bone Architecture, Strength, and Medullary Adipose Composition. In Department of Kinesiology, vol. Doctorate. Temple University.

[b7] Butler T. Pole A. Lunny M. Yingling V. 2013 Food restriction postpuberty is positive for bone structure long term. Med. Sci. Sports Exerc.; 45:105-107.

[b8] Bye A. Hoydal M. A. Catalucci D. Langaas M. Kemi O. J. Beisvag V. 2008 Gene expression profiling of skeletal muscle in exercise‐trained and sedentary rats with inborn high and low VO_2max_. Physiol. Genomics; 35:213-221. 18780757 10.1152/physiolgenomics.90282.2008 PMC2585023

[b9] Capparelli C. Whitaker‐Menezes D. Guido C. Balliet R. Pestell T. G. Howell A. 2012 CTGF drives autophagy, glycolysis and senescence in cancer‐associated fibroblasts via HIF1 activation, metabolically promoting tumor growth. Cell Cycle; 11:2272-2284. 22684333 10.4161/cc.20717 PMC3383589

[b10] Cerletti M. Jang Y. C. Finley L. W. Haigis M. C. Wagers A. J. 2012 Short‐term calorie restriction enhances skeletal muscle stem cell function. Cell Stem Cell; 10:515-519. 22560075 10.1016/j.stem.2012.04.002 PMC3561899

[b11] Chan G. K. Duque G. 2002 Age‐related bone loss: old bone, new facts. Gerontology; 48:62-71. 11867927 10.1159/000048929

[b12] Civitarese A. E. Carling S. Heilbronn L. K. Hulver M. H. Ukropcova B. Deutsch W. A. 2007 Calorie restriction increases muscle mitochondrial biogenesis in healthy humans. PLoS Med.; 4:e76 17341128 10.1371/journal.pmed.0040076 PMC1808482

[b13] Cohen H. Y. Miller C. Bitterman K. J. Wall N. R. Hekking B. Kessler B. 2004 Calorie restriction promotes mammalian cell survival by inducing the SIRT1 deacetylase. Science; 305:390-392. 15205477 10.1126/science.1099196

[b15] Cooper C. 1997 The crippling consequences of fractures and their impact on quality of life. Am. J. Med.; 103:17S-19S. 10.1016/s0002-9343(97)90022-x 9302893

[b16] Demontiero O. Vidal C. Duque G. 2012 Aging and bone loss: new insights for the clinician. Ther. Adv. Musculoskelet. Dis.; 4:61-76. 22870496 10.1177/1759720X11430858 PMC3383520

[b17] DiGirolamo D. J. Kiel D. P. Esser K. A. 2013 Bone and skeletal muscle: neighbors with close ties. J. Bone Miner. Res.; 28:1509-1518. 23630111 10.1002/jbmr.1969 PMC4892934

[b18] Du J. Klein J. D. Hassounah F. Zhang J. Zhang C. Wang X. H. 2014 Aging increases CCN1 expression leading to muscle senescence. Am. J. Physiol. Cell Physiol.; 306:C28-C36. 24196529 10.1152/ajpcell.00066.2013 PMC3919975

[b19] Edgar R. Domrachev M. Lash A. E. 2002 Gene Expression Omnibus: NCBI gene expression and hybridization array data repository. Nucleic Acids Res.; 30:207-210. 11752295 10.1093/nar/30.1.207 PMC99122

[b20] Hamrick M. W. 2011 A role for myokines in muscle‐bone interactions. Exerc. Sport Sci. Rev.; 39:43-47. 21088601 10.1097/JES.0b013e318201f601 PMC3791922

[b21] Hittel D. S. Kraus W. E. Tanner C. J. Houmard J. A. Hoffman E. P. 2005 Exercise training increases electron and substrate shuttling proteins in muscle of overweight men and women with the metabolic syndrome. J. Appl. Physiol. (1985); 98:168-179. 15347626 10.1152/japplphysiol.00331.2004

[b22] Huang D. W. Sherman B. T. Lempicki R. A. 2008 Systematic and integrative analysis of large gene lists using DAVID bioinformatics resources. Nat. Protoc.; 4:44-57. 10.1038/nprot.2008.211 19131956

[b23] Huang W. Sherman B. T. Lempicki R. A. 2009 Bioinformatics enrichment tools: paths toward the comprehensive functional analysis of large gene lists. Nucleic Acids Res.; 37:1-13. 19033363 10.1093/nar/gkn923 PMC2615629

[b24] Hunter G. R. McCarthy J. P. Bamman M. M. 2004 Effects of resistance training on older adults. Sports Med.; 34:329-348. 15107011 10.2165/00007256-200434050-00005

[b25] Joshi R. N. Safadi F. F. Barbe M. F. Del Carpio‐Cano F. Popoff S. N. Yingling V. R. 2011 Different effects on bone strength and cell differentiation in pre pubertal caloric restriction versus hypothalamic suppression. Bone; 49:810-818. 21807131 10.1016/j.bone.2011.07.019 PMC3772180

[b26] Kim B. Kim J. S. Yoon Y. Santiago M. C. Brown M. D. Park J. Y. 2013 Inhibition of Drp1‐dependent mitochondrial division impairs myogenic differentiation. Am. J. Physiol. Regul. Integr. Comp. Physiol.; 305:R927-R938. 23904108 10.1152/ajpregu.00502.2012

[b27] Kivela R. Kyrolainen H. Selanne H. Komi P. V. Kainulainen H. Vihko V. 2007 A single bout of exercise with high mechanical loading induces the expression of Cyr61/CCN1 and CTGF/CCN2 in human skeletal muscle. J. Appl. Physiol. (1985); 103:1395-1401. 17673559 10.1152/japplphysiol.00531.2007

[b28] Kodama Y. Umemura Y. Nagasawa S. Beamer W. G. Donahue L. R. Rosen C. R. 2000 Exercise and mechanical loading increase periosteal bone formation and whole bone strength in C57BL/6J mice but not in C3H/Hej mice. Calcif. Tissue Int.; 66:298-306. 10742449 10.1007/s002230010060

[b29] Krogh A. Larsson B. von Heijne G. Sonnhammer E. L. 2001 Predicting transmembrane protein topology with a hidden Markov model: application to complete genomes. J. Mol. Biol.; 305:567-580. 11152613 10.1006/jmbi.2000.4315

[b30] Kubota S. Takigawa M. 2007 Role of CCN2/CTGF/Hcs24 in bone growth. Int. Rev. Cytol.; 257:1-41. 17280894 10.1016/S0074-7696(07)57001-4

[b31] Kubota S. Takigawa M. 2011 The role of CCN2 in cartilage and bone development. J. Cell Commun. Signal.; 5:209-217. 21484188 10.1007/s12079-011-0123-5 PMC3145877

[b32] Layne J. E. Nelson M. E. 1999 The effects of progressive resistance training on bone density: a review. Med. Sci. Sports Exerc.; 31:25-30. 9927006 10.1097/00005768-199901000-00006

[b33] Lin S. J. Kaeberlein M. Andalis A. A. Sturtz L. A. Defossez P. A. Culotta V. C. 2002 Calorie restriction extends *Saccharomyces cerevisiae* lifespan by increasing respiration. Nature; 418:344-348. 12124627 10.1038/nature00829

[b34] Morales M. G. Gutierrez J. Cabello‐Verrugio C. Cabrera D. Lipson K. E. Goldschmeding R. 2013 Reducing CTGF/CCN2 slows down mdx muscle dystrophy and improves cell therapy. Hum. Mol. Genet.; 22:4938-4951. 23904456 10.1093/hmg/ddt352

[b35] Nishida T. Kubota S. Aoyama E. Janune D. Lyons K. M. Takigawa M. 2014 CCN family protein 2 (CCN2) promotes the early differentiation, but inhibits the terminal differentiation of skeletal myoblasts. J. Biochem.10.1093/jb/mvu056 10.1093/jb/mvu056 PMC6281389 25261584

[b36] Pedersen B. K. 2011 Muscles and their myokines. J. Exp. Biol.; 214:337-346. 21177953 10.1242/jeb.048074

[b37] Pedersen B. K. Febbraio M. A. 2012 Muscles, exercise and obesity: skeletal muscle as a secretory organ. Nat. Rev. Endocrinol.; 8:457-465. 22473333 10.1038/nrendo.2012.49

[b38] Petersen T. N. Brunak S. von Heijne G. Nielsen H. 2011 SignalP 4.0: discriminating signal peptides from transmembrane regions. Nat. Methods; 8:785-786. 21959131 10.1038/nmeth.1701

[b39] Pontius J. U. Wagner L. Schuler G. D. 2003 Unigene: A unified view of the transcriptome. In the NCBI handbook [Internet] Bethesda, MD National Center for Biotechnology Information (US)

[b40] Redman L. M. Rood J. Anton S. D. Champagne C. Smith S. R. Ravussin E. Pennington Comprehensive Assessment of Long‐Term Effects of Reducing Intake of Energy Research T. 2008 Calorie restriction and bone health in young, overweight individuals. Arch. Intern. Med.; 168:1859-1866. 18809812 10.1001/archinte.168.17.1859 PMC2748345

[b41] Rochard P. Rodier A. Casas F. Cassar‐Malek I. Marchal‐Victorion S. Daury L. 2000 Mitochondrial activity is involved in the regulation of myoblast differentiation through myogenin expression and activity of myogenic factors. J. Biol. Chem.; 275:2733-2744. 10644737 10.1074/jbc.275.4.2733

[b42] Safadi F. F. Xu J. Smock S. L. Kanaan R. A. Selim A. H. Odgren P. R. 2003 Expression of connective tissue growth factor in bone: its role in osteoblast proliferation and differentiation in vitro and bone formation in vivo. J. Cell. Physiol.; 196:51-62. 12767040 10.1002/jcp.10319

[b43] Schuler G. D. 1997 Pieces of the puzzle: expressed sequence tags and the catalog of human genes. J. Mol. Med. (Berl); 75:694-698. 9382993 10.1007/s001090050155

[b44] Schuler G. D. Boguski M. S. Stewart E. A. Stein L. D. Gyapay G. Rice K. 1996 A gene map of the human genome. Science; 274:540-546. 8849440

[b45] Sonnhammer E. L. von Heijne G. Krogh A. 1998 A hidden Markov model for predicting transmembrane helices in protein sequences. Proc. Int. Conf. Intell. Syst. Mol. Biol.; 6:175-182. 9783223

[b46] Tatsumi S. Ito M. Asaba Y. Tsutsumi K. Ikeda K. 2008 Life‐long caloric restriction reveals biphasic and dimorphic effects on bone metabolism in rodents. Endocrinology; 149:634-641. 17991723 10.1210/en.2007-1089

[b51] The UniProt Consortium. 2013 Update on activities at the Universal Protein Resource (UniProt) in 2013. Nucleic Acids Res.; 41:D43-D47. 23161681 10.1093/nar/gks1068 PMC3531094

[b47] Villareal D. T. Fontana L. Weiss E. P. Racette S. B. Steger‐May K. Schechtman K. B. 2006 Bone mineral density response to caloric restriction‐induced weight loss or exercise‐induced weight loss: a randomized controlled trial. Arch. Intern. Med.; 166:2502-2510. 17159017 10.1001/archinte.166.22.2502

[b48] Wang J. J. Ye F. Cheng L. J. Shi Y. J. Bao J. Sun H. Q. 2009 Osteogenic differentiation of mesenchymal stem cells promoted by overexpression of connective tissue growth factor. J. Zhejiang Univ. Sci. B; 10:355-367. 19434762 10.1631/jzus.B0820252 PMC2676415

[b49] Wheeler D. L. Church D. M. Federhen S. Lash A. E. Madden T. L. Pontius J. U. 2003 Database resources of the National Center for Biotechnology. Nucleic Acids Res.; 31:28-33. 12519941 10.1093/nar/gkg033 PMC165480

[b50] Zambon A. C. McDearmon E. L. Salomonis N. Vranizan K. M. Johansen K. L. Adey D. 2003 Time‐ and exercise‐dependent gene regulation in human skeletal muscle. Genome Biol.; 4:R61 14519196 10.1186/gb-2003-4-10-r61 PMC328450

